# Clinical phenotype and neuropathological correlates in autosomal dominantly inherited familial Alzheimer's disease: insights from a UK case series

**DOI:** 10.1002/alz70857_098875

**Published:** 2025-12-24

**Authors:** Natalie S Ryan, Clíona Farrell, Nanet Willumsen, Helen Rice, Moneeb Nasir, Xin Zhang, Charlotte Burdon, Dilek Ocal, Philip SJ Weston, Frances K Wiseman, Tammaryn Lashley, Nick C Fox

**Affiliations:** ^1^ Dementia Research Centre, UCL Queen Square Institute of Neurology, London, United Kingdom; ^2^ UK Dementia Research Institute at UCL, London, United Kingdom; ^3^ Queen Square Institute of Neurology, University College London, London, United Kingdom; ^4^ UK Dementia Research Institute, London, United Kingdom

## Abstract

**Background:**

Autosomal dominant familial Alzheimer's disease (ADAD), caused by *APP*, *PSEN1* and *PSEN2* mutations, is clinically and pathologically similar to sporadic AD, with both typically presenting with memory impairment. However as in sporadic AD, atypical phenotypes and neuropathological heterogeneity occur, including variability in the type of amyloid‐β plaque pathology, and in the degree of vascular amyloid‐β deposition as cerebral amyloid angiopathy (CAA). Investigating relationships between clinical, neuropathological and genetic heterogeneity in ADAD may provide insights into underlying pathophysiological and molecular mechanisms and inform strategies for targeting them therapeutically.

**Methods:**

We analyzed clinical phenotype data from over 250 symptomatic individuals from ADAD families seen at our Centre since the first mutation was identified over 30 years ago. 50 of these individuals underwent post‐mortem brain donation to the Institute of Psychiatry or Queen Square Brain Bank (QSBB); 37 with mutations in *PSEN1*, 13 in *APP*. Frontal cortex sections were stained immunohistochemically with a pan amyloid‐β antibody and vessel counts were used to determine the frequency and severity of CAA in leptomeninges and parenchyma. In the 29 cases donated to QSBB, patterns of amyloid‐β_40_, amyloid‐β_42_ and amyloid‐β_43_ antibody binding were also examined.

**Results:**

Age at onset was strongly influenced by the specific mutation but survival was influenced by mutation to a much lesser extent. Atypical (non‐amnestic) cognitive presentations with initial behavioral, language, dyscalculic or dysexecutive symptoms, and atypical clinical features (pyramidal, extrapyramidal and cerebellar signs) were more common in *PSEN1* than *APP* cases, particularly those with mutations post‐codon 200. In the post‐mortem cohort, 21 different mutations in *PSEN1*, four in *APP* and one *APP* duplication were represented. All cases demonstrated end‐stage AD pathology, however the frequency and severity of CAA varied considerably. Amyloid‐β_43_ deposition was seen in some individuals with *PSEN1* but not with *APP* mutations. Relationships between clinical and neuropathological features and patterns of amyloid‐β isoform binding were explored.

**Conclusions:**

Phenotypic heterogeneity in ADAD is influenced by causative gene and mutation type. Examining how mutation‐specific patterns of amyloid‐β peptide deposition relate to variability in CAA and clinical manifestations might inform the development of more personalised treatment approaches for individuals with ADAD mutations.

